# Clinical and Pathological Reports of Cases of Insanity

**Published:** 1884-04

**Authors:** S. V. Clevenger

**Affiliations:** Special Pathologist Cook County Hospital for the Insane


### ARTICLE IV.

### CLINICAL AND PATHOLOGICAL REPORTS OF CASES OF INSANITY.

By S. V. CLEVENGER, M.D.. Special Pathologist Cook County Hospital for the Insane.

On the first day of March, 1884, a general enumeration was made from the books of patients who were actually present on that day, with such matters pertaining to them as were of interest in their cases.

The new case books were begun in June last, when every one in the building was sought out and each case recorded, as far as information could be obtained. The numerical results of the work since then may be gathered from the following tables:

| | Males. | Females. | Total |
|---|---|---|---|
| Present June 1, 1883 | 227 | 273 | 500 |
| Admitted to March 1, 1884 | 152 | 161 | 313 |
| Total under observation | 379 | 434 | 813 |
| Of whom died | 35 | 41 | 76 |
| Of whom were discharged | 59 | 68 | 127 |
| Present March 1, 1884 | 285 | 325 | 610 |

Of whom 40 males and 26 females presented probably curable types of insanity.

| Ages. | Males. | Females. | Total. |
|---|---|---|---|
| Under 15 years | 1 | 2 | 3 |
| From 15 to 20 | 6 | 8 | 14 |
| " 20 to 30 | 102 | 96 | 198 |

| | | | |
|---|---|---|---|
| From 30 to 40 | 79 | 104 | 183 |
| " 40 to 50 | 63 | 67 | 130 |
| " 50 to 60 | 27 | 37 | 64 |
| " 60 to 70 | 5 | 9 | 14 |
| " 70 upwards | 2 | 2 | 4 |
| | 285 | 325 | 610 |

| Civil Condition. | Males. | Females. | Total |
|---|---|---|---|
| Married | 95 | 153 | 248 |
| Single | 172 | 126 | 298 |
| Widowed | 11 | 21 | 32 |
| Unknown | 7 | 25 | 32 |
| | 285 | 325 | 610 |

| Years when admitted. | Males. | Females. | Total. |
|---|---|---|---|
| 1863 | ... | 1 | 1 |
| 1866 | ... | 5 | 5 |
| 1867 | 2 | ... | 2 |
| 1868 | 1 | 1 | 2 |
| 1869 | 2 | 3 | 5 |
| 1870 | 3 | 2 | 5 |
| 1871 | 7 | 9 | 16 |
| 1872 | 4 | 2 | 6 |
| 1873 | 5 | 13 | 18 |
| 1874 | 5 | 2 | 7 |
| 1875 | 3 | 7 | 10 |
| 1876 | 7 | 14 | 21 |
| 1877 | 6 | 9 | 15 |
| 1878 | 12 | 16 | 28 |
| 1879 | 7 | 14 | 21 |
| 1880 | 9 | 20 | 29 |
| 1881 | 11 | 24 | 35 |
| 1882 | 51 | 40 | 91 |
| 1883 | 113 | 110 | 223 |
| 1884 | 37 | 33 | 70 |
| | 285 | 325 | 610 |

| Nativities. | Male. | Female. | Total. |
|---|---|---|---|
| American | 81 | 67 | 148 |
| Bohemian | 5 | 8 | 13 |
| Canadian | 6 | 3 | 9 |
| Dane | ... | 1 | 1 |
| Dutch | 3 | ... | 3 |
| English | 10 | 10 | 20 |
| French | 2 | 2 | 4 |
| German | 69 | 72 | 141 |
| Irish | 57 | 103 | 160 |
| Italian | 2 | ... | 2 |
| Norse | 12 | 11 | 23 |
| Pole | 4 | 8 | 12 |
| Scotch | 3 | 1 | 4 |
| Swede | 30 | 37 | 67 |
| Swiss | 1 | ... | 1 |
| Welsh | ... | 1 | 1 |
| Unknown | ... | 1 | 1 |
| | 285 | 325 | 610 |

| | Foreign Born. | | Native Born. | | | | |
|---|---|---|---|---|---|---|---|
| Races. | Males. | Females. | Males. | Females. | Total. Males. | Total. Females. | Total. |
| Anglo Saxon | 21 | 14 | 33 | 39 | 54 | 53 | 107 |
| Celtic | 56 | 103 | 29 | 15 | 85 | 118 | 203 |
| Germanic | 67 | 63 | 12 | 4 | 79 | 67 | 146 |
| Hebrew | 4 | 7 | ... | 1 | 4 | 8 | 12 |
| Latin | 6 | 2 | 1 | ... | 7 | 2 | 9 |
| Negro | ... | 1 | 4 | 8 | 4 | 9 | 13 |
| Scandinavian | 42 | 49 | ... | ... | 42 | 49 | 91 |
| Sclavonic | 10 | 19 | ... | ... | 10 | 19 | 29 |
| | 206 | 258 | 79 | 67 | 285 | 325 | 610 |

SUMMARY.

| | Ma'es. | Females. | Total. |
|---|---|---|---|
| Native born Americans | 33 | 39 | 72 |
| Native and Foreign, other nationalities | 252 | 286 | 538 |
| | 285 | 325 | 610 |

| Occupation. | Males. |
| --- | --- |
| Trades requiring skillfulness | 114 |
| Occupations in which skill is not required | 134 |
| Merchants | 6 |
| Professional | 3 |
| No occupation | 28 |
| | 285 |

| Occupation. | Females. |
| --- | --- |
| Housewives | 142 |
| Domestics | 87 |
| Seamstresses | 12 |
| Actresses | 7 |
| Teachers | 4 |
| Clairvoyant | 1 |
| Unknown | 77 |
| | 325 |

In the list of psychoses Spitzka's classification has been adhered to as the most scientific one in existence. America has reason to be proud of having originated an alienist who has brought order out of chaos in these matters, and the deeper the asylum physician delves into his cases and compares them with Spitzka's work on insanity, the more will he appreciate the clearness and profundity of the classification as well as the entire subject matter of his book.

I have found it convenient to designate acute from chronic forms of mania, melancholia, etc., as the terms possess something of a prognostic value. In former clinical reports I mentioned monomania as a misnomer, and suggested that a name conveying the idea of logical perversion would be more appropriate for this disorder. Since then I have encountered the term paranoia, as used by Giuseppe, Amadei, and Silvio Tonnini, for this form of insanity, in the Nov. '83 leading article of the *Archivio Italiano per le Malattie Nervose e Alienazoni Mentali*, the organ of the Italian *Societa Freniatrica*, and in the expectation that it will come into general use instead of the word which has caused so much misunderstanding, have adopted it.

Such terminal dements as possess a modicum of their former mental troubles I have separated off into secondary confusional insane as Spitzka suggests. It forms an excellent link between the chronic and terminal types of insanity.

PSYCHOSES.

| | Males. | Females. | Total. |
| --- | --- | --- | --- |
| Acute alcoholic insanity | 4 | 1 | 5 |
| Acute hysterical insanity | ... | 1 | 1 |
| Acute mania | 12 | 10 | 22 |
| Acute melancholia | 15 | 10 | 25 |
| Chronic alcoholic insanity | 15 | 3 | 81 |

| | | | |
|---|---|---|---|
| Chronic hysterical insanity | ... | 12 | 12 |
| Choreic insanity | 1 | ... | 1 |
| Circular insanity | 3 | ... | 3 |
| Chronic mania | 25 | 40 | 65 |
| Chronic melancholia | 16 | 25 | 41 |
| Epileptic insanity | 31 | 20 | 51 |
| Hebephrenia | 9 | 7 | 16 |
| Idiocy | 1 | ... | 1 |
| Imbecility | 6 | 8 | 14 |
| Katatonia | 10 | 3 | 13 |
| Paranoia | 16 | 6 | 22 |
| Paretic dementia | 13 | 2 | 15 |
| Primary confusional insanity | 6 | 3 | 9 |
| Recurrent mania | 4 | 13 | 17 |
| Recurrent melancholia | 1 | 2 | 3 |
| Secondary confusional insanity | 28 | 60 | 88 |
| Senile dementia | 5 | 7 | 12 |
| Stuporous insanity | 3 | ... | 3 |
| Syphilitic insanity | 2 | ... | 2 |
| Terminal dementia | 59 | 91 | 150 |
| Transitory frenzy | ... | 1 | 1 |
| | 285 | 325 | 610 |

The medical Aristarchus can easily find fault with any ætiological table, and particularly where insanity is the effect produced. It is extremely difficult to arrive at probable causes in the main, from many reasons; among them being the absolute want of all information concerning the insane sent to asylums, (aside from such cases where nothing is known by those who send them here, this is an outrage unworthy even barbarians, for often a hint from friends will materially assist toward the treatment and care of the insane); also the disposition, occasionally met with, to hold information back from the physicians from ignorant motives on the part of relatives, and the general illogicality of the masses.

A large proportion of the insane are sent to us as masturbatory; this and religious excitement are main alleged causes. Both may be repudiated as being in 99 per cent. of cases *effects* of the insanity, and not causes. In some of the cases present I have noticed that hernia not only accompanies the disorder, but is at least an aggravator of the mental derangement, if not a direct cause. Certainly, where coprostasis causes melancholia, as grave an intestinal trouble as rupture may also be reckoned ætiological. In male case No. 123, with complete scrotal hernia, and the delusion that he is to be hanged every day at 10 A. M., it is noticeable that when confined to bed on account of the tumor, his mental condition is much worse. The hernias may be allowed to remain, at least, as tentatively ætiological.

Although alcoholism is an effect as well as a cause of insanity, in my cases, I incline to the belief that the estimate is too low.

English statistics place it at one-half of all cases; French and Russian somewhat lower. Were the exact histories known of those concerning whom nothing could be obtained, fully 30 per cent. of insanity in males would, in my opinion, thus be caused.

Besides this, while alcoholism, as might be expected, is small among females, insanity is in many cases induced in them by the indirect effects of the bane in husbands, fathers, brothers, etc. For example, drunken husbands abuse, starve, overwork, and desert their wives, and one case shows insanity to date from a drunken brother striking his sister on the head with a bottle.

Though anæmia may be due to multiple causes, it may properly be classed as causative, and menorrhagia may be also included as inducing anæmia, though placed among menstrual derangements.

I think that puerperal troubles would show a larger percentage were the histories of old cases obtainable in every instance, and from various reasons, I incline to the belief that most puerperal cases, if not all, were predisposed by heredity. In fact, hereditary taint plays a very important part in mental alienation, and some very interesting deductions may be made from the following tables, which I have preferred to give fully, in spite of minor objections which may be raised against various facts, such as paralysis being accredited as a cause in one case. It may merely have accompanied the insanity, and while we do not know what caused either the insanity or the paralysis, it is safe to assume a cerebral blood vessel had ruptured, and the word paralysis sufficiently points to that probability.

PROBABLE CAUSES OF INSANITY.

SIMPLE PROBABLE CAUSES.

| Moral causes. | Males. | Females. | Total. |
|---|---|---|---|
| Abuse by husband | ... | 5 | 5 |
| Domestic trouble | ... | 11 | 11 |
| Fright | 3 | 4 | 7 |
| Grief | 6 | 6 | 12 |
| Love disappointment | ... | 2 | 2 |
| Mental overstrain | 1 | 1 | 2 |
| Nostalgia | 1 | 1 | 2 |
| Poverty | 2 | 1 | 3 |
| Seduction | ... | 1 | 1 |
| Physical causes. | | | |
| Alcoholism | 43 | 12 | 46 |
| Acquired diseases, etc. | | | 5 |
| Anæmia | 5 | ... | 1 |
| Chorea | 1 | ... | 3 |
| Epilepsy | 24 | 15 | 9 |
| Erysipelas | ... | 1 | 1 |
| "Fevers" | 1 | ... | 1 |

| | | | |
|---|---|---|---|
| Hernia | 2 | ... | 2 |
| Heart disease | 1 | ... | 1 |
| Hysteria | ... | 5 | 5 |
| Locomotor ataxia | 1 | ... | 1 |
| Meningitis | 1 | 1 | 2 |
| Measles | 1 | ... | 2 |
| Menstrual derangements, Amenorrhœa | ... | 2 | 5 |
| " " Dysmenorrhœa | ... | 5 | 1 |
| " " Menorrhagia | ... | 1 | 1 |
| Over lactation | ... | 4 | 4 |
| Overwork | ... | 3 | 1 |
| Puerperal | ... | 20 | 20 |
| Paralysis | 1 | ... | 1 |
| Puberty | 2 | 4 | 5 |
| Rheumatism | 2 | ... | 2 |
| Rachitis | ... | 1 | 1 |
| Senility | 4 | 7 | 11 |
| "Sickness" | 1 | 7 | 3 |
| Syphilis | 2 | ... | 2 |
| Smallpox | 1 | ... | 1 |
| Spinal caries | ... | 1 | 1 |
| Scarlatina | 1 | ... | 1 |
| Scrofula | 1 | 1 | 2 |
| Traumatism, Head injuries | 23 | 3 | 26 |
| " Insolation | 3 | 1 | 4 |
| " Intense heat | 2 | ... | 2 |
| " Lightning stroke | 1 | ... | 1 |
| Typhoid fever | 3 | 1 | 4 |
| Typhus fever | ... | 1 | 1 |
| Inherited Insanity | 14 | 26 | 40 |
| " Congenitally defective mentality | 3 | 3 | 6 |
| " Congenital rachitis | ... | 1 | 1 |
| " Diseases in general | 2 | 1 | 3 |
| " Syphilis | 1 | ... | 1 |
| " Epilepsy | 1 | ... | 1 |

COMPLEX PROBABLE CAUSES.

| | Males. | Females. | Total. |
|---|---|---|---|
| Alcoholism and Syphilis | 1 | ... | 1 |
| " " Intense heat | 1 | .. | 1 |
| " " Hernia | 1 | ... | 1 |
| " " Heredity and traumatism | 2 | ... | 2 |
| " " Heredity | 1 | 1 | 2 |
| " " Opiophagism | ... | 1 | 1 |
| " " Traumatism | 1 | ... | 1 |
| " " Epilepsy | 1 | ... | 1 |
| " " Epilepsy and hernia | 1 | ... | 1 |
| Congenital and scarlatina | ... | 1 | 1 |
| Epilepsy, inherited scrofula, and trychophytosis | 1 | ... | 1 |
| " and puerperal | ... | 2 | 2 |
| " " Puberty | ... | 1 | 1 |
| " " Overwork | ... | 1 | 1 |
| " " Puberty and heredity | 1 | .... | 1 |
| " " Traumatism | 3 | ... | 3 |
| Traumatism and Typhoid fever | 2 | ... | 2 |
| " " Insolation | 1 | ... | 1 |
| " " Puberty | 1 | 1 | 2 |
| " " Fright | 1 | .... | 1 |
| Heredity and Traumatism | .... | 1 | 1 |
| " " Love disappointment | 1 | 1 | 2 |
| " " Senility | 1 | 1 | 2 |
| " ' Insolation | ... | 1 | 1 |
| " " Puerperal | .... | 4 | 4 |
| " " Dysmenorrhœa | ... | 1 | 1 |
| Heredity and puberty | 1 | .... | 1 |
| Puberty and scarlatina | 1 | .. | 1 |
| Hysteria and love disappointment | .... | 1 | 1 |
| Senility and hernia | .... | 1 | 1 |
| Sickness and exposure | ... | 1 | 1 |
| Scrofula and hysteria | .. | 1 | 1 |
| Typhoid fever and hernia | ... | 2 | 2 |
| | 175 | 182 | 357 |
| Unascertained | 100 | 143 | 253 |
| | 285 | 325 | 610 |

PROBABLE CAUSES CONDENSED.

| | Simple | | With Complications | | |
|---|---|---|---|---|---|
| | Males. | Females. | Males. | Females. | Total. |
| Total moral causes | 13 | 32 | 2 | 2 | 49 |
| " Acquired diseases, etc | 49 | 37 | 13 | 18 | 117 |
| " Traumatism | 29 | 4 | 13 | 3 | 49 |
| " Puerperal and lactational | ... | 24 | ... | 6 | 30 |
| " Alcoholism | 34 | 12 | 9 | 2 | 57 |
| " Menstrual derangements | .... | 8 | .... | 1 | 9 |
| " Inherited insanity | 14 | 26 | 7 | 10 | 57 |
| " " diseases | 7 | 5 | 1 | 1 | 14 |
| " Senility | 4 | 7 | 1 | 3 | 15 |
| " Puberty | 2 | 4 | 5 | 2 | 13 |
| Total causes | 152 | 159 | 51 | 48 | 420 |
| Operative in cases | 152 | 159 | 23 | 23 | 357 |

CAUSES PER CENT. ASCERTAINED PROBABLE.

| | Males. | Females. | Total. |
|---|---|---|---|
| Alcoholism | 22 | 6 | 14. |
| Acquired diseases | 28 | 24 | 26. |
| Traumatism | 21 | 3 | 12. |
| Puerperal and lactational | .... | 15 | 7.5 |
| Menstrual derangements | ... | 4 | 2. |
| Moral causes | 8 | 19 | 13.5 |
| Inherited insanity | 12 | 19 | 15.5 |
| " diseases | 3 | 2 | 2.5 |
| Senility | 2 | 5 | 3.5 |
| Puberty | 4 | 3 | 3.5 |
| | 100 | 100 | 100 |
| Insanity largely due to inherited defects | 29 | 67 | 48 |
| " " " acquired defects | 71 | 33 | 52 |

An important fact seems to have been elicited by the researches embodied in the foregoing tables, and that is: *Females largely inherit their insanity, while males largely acquire theirs.*

The statistics of single asylums must be taken upon their own merits, and many things considered in connection with them, but it is worth consideration that, so far as I have been able to ascertain, fully one-half the insanity in this asylum has been transmitted by diseased parents, and that the males are more apt to become insane without heredity, while the females are more apt to have inherited their mental defects, or this may be read in another way, could the statistics outside of asylums be obtainable: the male is less apt to succumb to hereditary taint than the female, nevertheless the italicized deduction above may remain as it is.

